# Plant-Based Diets and Peritoneal Dialysis: A Review

**DOI:** 10.3390/nu14061304

**Published:** 2022-03-19

**Authors:** Scott E. Liebman, Shivam Joshi

**Affiliations:** 1 Department of Medicine, Division of Nephrology, University of Rochester School of Medicine and Dentistry, Rochester, NY 14642, USA; 2 Department of Medicine, New York University Grossman School of Medicine, New York, NY 10016, USA; shivam.joshi@nyulangone.org; 3 Department of Medicine, NYC Health + Hospitals/Bellevue, New York, NY 10016, USA

**Keywords:** peritoneal dialysis, plant-based diets, nutrition

## Abstract

Whole food plant-based diets are gaining popularity as a preventative and therapeutic modality for numerous chronic health conditions, including chronic kidney disease, but their role and safety in end-stage kidney disease patients on peritoneal dialysis (PD) is unclear. Given the general public’s increased interest in this dietary pattern, it is likely that clinicians will encounter individuals on PD who are either consuming, considering, or interested in learning more about a diet with more plants. This review explores how increasing plant consumption might affect those on PD, encompassing potential benefits, including some specific to the PD population, and potential concerns.

## 1. Introduction

Whole food plant-based diets (WFPBD: a dietary pattern that focuses on unprocessed foods derived from plant sources (fruits, vegetables, whole grains, and legumes) while avoiding animal-based products (meat, fish, dairy, and eggs) and processed plant products (including vegetable oils and most pre-cooked, pre-packaged products sold in grocery stores)) are increasingly recommended for the prevention and management of conditions such as obesity [1], hypertension [2,3], diabetes [4,5], and cardiovascular disease [6,7,8]. There is also increasing evidence that plant-based diets may be beneficial in the prevention [9] and management of chronic kidney disease [10,11,12]. Whether plant-based diets are advantageous in individuals with end-stage kidney disease (ESKD), particularly those on peritoneal dialysis (PD), is unclear.

As of 2018, there were approximately 58,500 individuals on PD in the United States, representing about 10.6% of the total dialysis population [13]. Those on PD have a high mortality rate and a large burden of comorbidities, especially cardiovascular disease [13]. Data from the United States Renal Data System shows 1-, 3-, and 5-year mortality rates for people on peritoneal dialysis to be 10%, 32.4%, and 53.1%, respectively, with cardiovascular disease (CVD) accounting for 41% of deaths [13]. The predominance of CVD (amenable to amelioration with a WFPBD), as a cause of mortality, and the high prevalence of risk factors such as hypertension and diabetes (also amenable to amelioration with a WFPBD) in PD patients [13,14] suggests that dietary intervention may be a strategy to reduce mortality in the PD population.

To date, data on plant-based diets are limited in the PD population, and the interpretation of existing data in any population is complicated by a lack of consistency in the use of the term “plant-based”. They may differ as to the degree of the plant-based component (i.e., totally vs. predominately plant-based, with varying amounts of animal products) and the degree to which the diets consist of whole foods (some diets may be completely plant-based or vegan but contain a large amount of processed foods). Nonetheless, PD patients have several common comorbidities in which a WFPBD may theoretically be of benefit.

Peritonitis is arguably the most concerning complication of PD. Despite a decreasing incidence, peritonitis remains the most common cause of technique failure among individuals using PD [15], and it confers a significant risk of hospitalization and mortality [16]. One potential source of peritonitis in PD patients is the enteric translocation of organisms. While there are currently no data on the association between dietary patterns and peritonitis risk, one can speculate that WFPBDs may offer some protection from this particular route of infection due to beneficial effects on the gut microbiome and dysbiosis. Constipation, fiber deficiency, and hypokalemia are common in PD patients, and eating more plants can help ameliorate all of these, as will be discussed below.

As the popularity of and interest in plant-based eating increases [17,18], it is likely that clinicians will encounter individuals using PD who are either consuming, considering, or simply interested in this dietary pattern. This narrative review explores the current state of knowledge and reviews the potential benefits and concerns of plant-based diets and increasing plant intake in the PD population.

## 2. Potential Benefits of Plant-Based Diets in the Peritoneal Dialysis Population

### 2.1. Mortality

To date, no studies have examined the effect of eating a completely WFBPD compared with other dietary patterns on mortality in the ESKD population. In hemodialysis (HD) patients, an increased fruit and vegetable intake (although still within the context of continued consumption of animal products) is associated with a decrease in all-cause mortality [19].

In peritoneal dialysis patients, the data are also limited to an analysis of the degree of plant-based eating (within a dietary pattern that also contains animal products), rather than a completely WFPBD per se. In a retrospective study of 884 Chinese peritoneal dialysis patients, those in the highest tertile of plant protein intake (>57.5% of protein from plant sources) had a 24% decrease in mortality compared to the lowest tertile (<47.7% of protein from plant sources), despite their absolute total protein intake being lower (48.7 vs. 54.7 g per day) [20]. A subgroup analysis in this study showed that not all subjects achieved this benefit. Specifically, the mortality benefit was seen in females, those over 60 years old, and those with a baseline albumin of >3.5 g/dL. This suggests that eating more plants (and hence, more plant-based protein) may help mitigate mortality in the PD population. Prospective (ideally randomized) studies would be needed to further test this hypothesis and to determine which subgroups would derive this benefit.

### 2.2. Volume Overload and Sodium Intake

Volume overload is common in individuals on PD. The International Society of Peritoneal Dialysis cardiovascular and metabolic guidelines consider its assessment a “vital component in the management of PD patients” [21]. Volume overload decreases survival. In a cohort study of >1000 patients, Van Biesen et al. [22] demonstrated via bioimpedance spectroscopy that volume overload was common upon starting PD and, although improved, persisted even after three years of dialysis, with a mean degree of volume overload of 7.7%. Further analysis showed that those above the 75th percentile of volume overload at one month had a 59% increased risk of mortality [22].

One of the mainstays in controlling volume overload in the PD population is sodium restriction. Günal et al. [23] demonstrated that many PD patients could improve volume status and achieve blood pressure control via meticulous attention to sodium intake (via a “salt-poor diet and not using ready-made food”). Plant-based diets are often lower in sodium than other dietary patterns. Several studies have shown that vegans consume less than half the amount of sodium than that of the reference group of either omnivores [24] or the general population [25]. Given this, one can speculate that a nutritionally adequate plant-based diet may be a beneficial strategy in controlling volume overload in individuals on PD with the caveats that (a) it is possible to eat a high sodium plant-based diet if one relies heavily on processed foods (including meat analogs, which are increasingly available in grocery stores) and (b) a lower sodium diet that comes at the expense of adequate protein or energy intake, which should be avoided as it may increase mortality in the PD population [26]. Finally, it should be noted that plant foods have a higher water content than non-plant foods and may require a reduction in free fluid intake to maintain euvolemia.

### 2.3. Constipation/Fiber

Constipation is a common problem for those on PD and may have serious consequences due to its effect on the dialysate flow and an increased risk of peritonitis [27]. Although constipation is a multifactorial process, a deficiency of dietary fiber is a major contributing factor. People on PD on average consume between 8 and 9 g of fiber per day [28,29], both well short of that recommended by the Institute of Medicine (25 or 38 g for women and men 19–50 years old, respectively, and 21 or 30 g for those over the age of 50) [30], As such, increasing dietary fiber has been recommended as a first-line therapy for patients with constipation on PD [27], and studies have shown improvement in constipation with this approach [31,32,33].

Although the dietary fiber intake can be increased using supplements, plant-based diets are naturally high in fiber and may also be used to treat constipation. Since fiber is exclusively found in plant-foods, it is not surprising that those consuming plant-based diets have significantly higher daily intakes of fiber compared to those not following this diet plan [34,35], with one study showing that vegans consume 74% more fiber than non-vegetarians [34].

In addition to its role in improving constipation, increased fiber intake may offer other beneficial effects in individuals on peritoneal dialysis. A cross-sectional study showed that those with dietary fiber intake >12.2 g per day had a lower concentration of inflammatory markers in both serum and dialysate [28]. In a cohort study of 881 peritoneal dialysis patients, those in the middle or highest tertile of dietary fiber intake (although still low at 7.8 and 11.8 g/day) showed an increase in albumin over time compared with the lower group [29]. There was also a trend toward increased mortality in the lowest tertile, although this did not achieve significance. From these studies, however, it is not possible to conclude whether fiber *per se* was offering these benefits or another aspect of consuming higher fiber diets, and further studies would be needed to determine this.

### 2.4. Gut Microbiome

The human gut microbiome contains trillions of bacteria, along with viruses, fungi, and archaea [36]. The microbiome exists in symbiosis with the host, and it provides many key functions, including those related to immunity; endocrine function; energy biogenesis; biosynthesis of vitamins, steroid hormones, and neurotransmitters; and the metabolism of dietary components, drugs, and branched chain aromatic amino acids [36]. While there is no “gold standard” as to what constitutes a healthy gut microbiome, differences have been noted between healthy individuals and those with a variety of disease states, including chronic kidney disease, and those on dialysis [36,37]. These differences include a decrease in overall diversity and a change in the composition of the microbiome, with different phyla being either more or less represented [37,38,39]. Collectively, this imbalance in the composition and function of the intestinal microbiota is known as dysbiosis, and it is associated with negative consequences to the host [36]. One hypothesized negative effect of dysbiosis is an alteration of the gut epithelial integrity (“leaky gut” or “leaky mucosa”), which may lead to the translocation of bacteria or inflammatory products such as endotoxins [38]. This is directly relevant to individuals on peritoneal dialysis. PD patients have high plasma levels of bacterial-derived fragments [40] and endotoxins [41,42], and some studies have shown an association between these factors and higher rates of inflammation [42,43] and pointedly cardiovascular disease [40,42], which, as noted, earlier is the number one cause of mortality in this population. Changes in gut epithelial integrity are also a concern given the not uncommon occurrence of PD-related enteric peritonitis. Dysbiosis also leads to increased production of the uremic toxins indoxyl sulfate (IS) and p-cresol/p-cresyl sulfate (PCS) [44], which have been associated with progression of kidney disease [45] in those with chronic kidney disease (CKD). Prospective studies have not been done evaluating the role of IS and PCS in individuals on PD, but these data are concerning given the importance of residual kidney function (RKF) in this population. IS and/or PCS have been shown to rise concurrently with RKF loss in those on PD [46] and have been associated with other deleterious outcomes, including technique failure, cardiac events, and mortality [47].

Diet is one of many factors which can affect the microbiome. Vegans have distinctly different microbiomes than due omnivores, whereas the data comparing vegans and vegetarians are less clear [48]. It has not been definitively demonstrated that switching diets to change microbiome composition leads to lasting health benefits. Short term, the microbiome appears to be resilient to dietary intervention, reverting back to its core composition once the intervention ends [49]. Nonetheless, there are data which suggest that a consistent plant-based diet may confer benefits via the microbiome. A small interventional study in which obese subjects ate a vegan diet demonstrated (in addition to improvement in weight, triglycerides, total cholesterol, low-density lipoprotein (LDL)-cholesterol, and hemoglobin A1c) a reduction in the number of pathobionts (organisms that cause harm only under certain circumstances (such as *Enterobacteriaceae*) [50]. Again, this may be directly relevant in PD, where this class of bacteria may cause peritonitis. The investigators also found a decrease in inflammatory markers [50].

Another postulated benefit of plant-based diets mediated by the microbiome is a decreased production of trimethylamine-N-oxide (TMAO), which mediates atherosclerosis. Vegetarians and vegans have decreased baseline levels of TMAO compared with omnivores, and they produced less of it when challenged with L-carnitine [51]. The potential implication of this is suggested by a recent study showing an association between TMAO with all-cause (all subjects) and cardiovascular mortality (male subjects) in individuals on PD [52].

The microbiome also produces short chain fatty acids (SCFA), including acetate, propionate, and butyrate [53]. Previous research has shown that children consuming a more traditional, plant-based diet produce more SCFA than those on a more Western diet [54]. SCFAs are anti-inflammatory and have a host of beneficial effects, including improved gut epithelial integrity, blood pressure regulation, and improved lipid and glucose homeostasis [53], all of which would be beneficial to the PD population.

Further research on the effects of diet on the microbiome and the microbiome in general is needed in PD patients.

### 2.5. Hypertension

Hypertension is common in those with ESKD, although the exact prevalence is difficult to determine given the different definitions, techniques, and settings of measurements. Several studies demonstrate that 70–80% of individuals on dialysis (including both HD and PD) are hypertensive and that the majority are uncontrolled [14,55,56]. Plant-based diets are effective in treating hypertension in the general population [2,57], and there is evidence that they benefit hypertensive CKD patients as well. In two separate randomized controlled trials, Goraya et al. [10,11] studied the effect of adding fruits and vegetables to the diets of individuals with CKD 3 and 4 and acidosis. In hypertensive patients with CKD 3 or CKD 4, those whose acidosis was treated with fruits and vegetables had the added benefit of blood pressure reduction when compared with those treated with bicarbonate (CKD 3 and CKD 4 patients) or placebo (CKD 3 patients). As noted above, Günal et al. [23] were able to achieve good hypertension control with a salt-restricted diet, but the dietary pattern(s) consumed were not clear. There have been no randomized controlled trials or cohort studies to date examining the relationship between fruit and vegetable intake or a diet higher in plants and hypertension in individuals on PD.

### 2.6. Metabolic Acidosis

Metabolic acidosis is common in patients with CKD, and its prevalence increases with CKD severity [58]. The consequences of metabolic acidosis include bone disease, muscle protein catabolism, decreased albumin synthesis, and increased inflammation [59]. Retrospective cohort studies have demonstrated an association between the dietary acid load or degree of metabolic acidosis and worsening kidney function in those with CKD [60,61,62].

Treating metabolic acidosis with sodium bicarbonate preserves kidney function in individuals with CKD [63], but one concern with using pharmacologic bicarbonate is the sodium load. Adjusting the acid content via changes in diet can mitigate this problem. Dietary manipulation requires an appreciation of the acidogenic potential of different foods. On the whole, animal-derived foods such as cheese, meat, and fish tend to be highly acidogenic, whereas plant-based foods tend be less so, with some grains (particularly if highly processed) being an exception [64]. Fruits and vegetables deserve special mention, as they are not only less acidogenic than animal-based foods, but are actually alkaline or acid consuming [64].

In CKD patients, the treatment of metabolic acidosis with fruits and vegetables (in the context of a diet still containing animal protein) leads to a decrease in net acid excretion [65], a reduction in blood pressure and weight, and, in CKD 3 patients, a decrease in the rate of progression of kidney disease similar to that seen with bicarbonate [11].

The relationship between bicarbonate concentration to adverse outcomes is less well-defined in PD patients. A prospective study of >400 PD patients showed that those with a time-averaged serum bicarbonate <24 mEq/L had a higher risk of becoming anuric and of residual kidney function decline compared with those >24 mEq/L [66]. Two small randomized trials have examined using bicarbonate in PD patients with a bicarbonate level <24 mEq/L [67,68]. Both showed an improvement in acidosis. One trial showed a preservation of residual kidney function [67], whereas the other did not, although it did note improvement in nutritional status via the subjective global assessment score [68]. Studies comparing either a plant-based diet or the selective enhancement of fruit and vegetable consumption on acidosis correction and residual kidney function preservation as done in the CKD population have not been done in those on PD.

## 3. Potential Disadvantages of Plant-Based Diets in the Peritoneal Dialysis Population

### 3.1. Potassium/Hyperkalemia

One major concern with advocating plant-based diets in those on dialysis is the risk of hyperkalemia. Fear of hyperkalemia often results in advice to reduce dietary potassium, potentially depriving those with kidney disease of the cardiovascular benefits associated with increased potassium intake [69]. Several considerations may help mitigate this concern.

It is important to note that hypokalemia is not uncommon in those on PD [70,71,72]. An observational cohort study of >100,000 dialysis patients (including >10,000 on PD) found a 4.7-fold increased risk in hypokalemia for those on PD compared to those on HD [72]. Hypokalemia was shown to be a risk factor for all-cause, cardiovascular, and infection-related mortality in individuals on PD, and the increased risk for mortality with a K^+^ < 3.5 mEq/L was comparable to that with a K^+^ of 5.5 mEq/L or higher [72]. The increase in mortality, cardiovascular mortality, and infection-related mortality with hypokalemia was also demonstrated in a cohort of Brazilian individuals on PD, in which the authors used a propensity-matched score analysis as an attempt to reduce confounding [73].

Hypokalemia was shown to be a risk factor for peritonitis in a cohort of PD patients in Taiwan, particularly with bacteria of enteric origin [74]. The authors hypothesized that hypokalemia may lead to bowel dysmotility, may be a sign of overall malnutrition, and may be responsible for altering the immunologic defense, leading to translocation of bacteria from the gut into the peritoneum [74].

Whether or not correcting hypokalemia in PD patients leads to improved outcomes has not been evaluated, but it has been estimated that 10–29% of hypokalemic PD patients use potassium supplements [72]. In these patients, diets high in plant-based foods and rich in potassium may be an appropriate (albeit unproven) strategy to help improve serum potassium, while providing benefits such as fiber, alkali, and phytochemicals not found in supplements.

While augmenting dietary potassium in frankly hypokalemic patients, even if not helpful, is unlikely to be harmful, concern may remain for those with a normal serum potassium. Although the safety of plant-based diets has not be evaluated in individuals on PD with normal potassium, some data suggest that these concerns may be unwarranted.

While a plant-based diet is potassium rich, it is often overlooked that animal proteins, such as dairy and meat (especially organ meats), also are high in potassium [69]. Several studies show that the difference in the amount of potassium consumed by vegans and those following other dietary patterns (in the general population) is either not very large or non-existent [24,25,34]. Food additives may be a hidden source of potassium in animal products, surreptitiously increasing its intake, often dramatically [75]. Additives may be a particular concern in low sodium processed foods [76]. Another consideration is the method of cooking and consumption. Boiling fruits or vegetables decreases potassium content, whereas drying them or processing them into juices or sauces increases it [12,69].

Potassium bioavailability may be a consideration. The relationship between potassium intake and serum potassium levels in patients with ESKD on HD does not seem to be linear or robust. In a study of more than 8000 hemodialysis patients, dietary potassium was not associated with serum potassium levels, hyperkalemia, or either cardiac or all-cause mortality [77]. Similarly in a secondary analysis of the Nutritional Inflammatory Evaluation Study, pre-HD potassium levels were not significantly different between quartiles of K^+^ intake, and when potassium intake was examined as a continuous variable, the absolute difference in serum K^+^ between the highest and lowest dietary levels was only 0.4 mEq/L [69,78].

For those on PD, there are no studies specifically examining dietary potassium intake and serum potassium. In the study by Liu et al. noted above, there were no differences in serum potassium between the highest and lowest tertile of plant protein intake (again in the context of a diet containing animal products), although dietary potassium was not specifically assessed [20].

Importantly, the bioavailability of potassium changes with the form of potassium in the diet. In those with normal kidney function, eating foods processed such that cell walls are disrupted led to a 25% increase in potassium bioavailability [79]. Several studies in CKD suggest a differential bioavailability of potassium from plant protein and animal protein [80,81].

Again, studies in PD are limited. Blumenkrantz and colleagues [82] reported the results of metabolic balance studies in PD patients consuming diets differing in the amount of protein (1 g/kg/day vs. 1.4 g/kg/day) and potassium (64 mEq/day vs. 84 mEq/day). While the serum potassium values were not reported in the study, the authors commented in the discussion that “Serum potassium levels were normal or in the lower-range of normal in most patients. These findings were present despite the rather high potassium intake, particularly with the higher protein diet.” [82]. Taken together, the available data suggest that in CKD and ESKD patients, including those on PD, consuming a diet high in unprocessed plant foods may not lead to as great an increase in serum K^+^ as one might initially expect.

How might individuals with potentially severely compromised kidney function maintain normal potassium levels despite increased dietary intake? Alkalemia and insulin tend to promote the cellular uptake of potassium, and the consumption of plant food, particularly fruits and vegetables, may induce both insulin secretion and a more alkalemic (or at least less acidemic) environment [83]. Although the cellular uptake of K^+^ may temporize, absorbed K^+^ must eventually be excreted to maintain balance. In individuals without CKD, the kidneys excrete most of the absorbed potassium. In individuals with dialysis-dependent ESKD, dialysis obviously is a major source of K^+^ removal. CKD patients (receiving dialysis or not) can also augment colonic K^+^ loss. Early balance studies by Hayes et al. showed that as kidney function worsens, the ability of the colon to excrete potassium in the face of an increased dietary load was higher in those with kidney disease than those without [84]. HD patients were able to increase stool potassium by 3-fold or more compared with normal controls [84]. It is not clear whether PD patients can augment fecal potassium excretion similarly to those on HD, although Blumenkrantz et al. [82] found that PD patients do seem to increase stool K^+^ excretion to some degree in the face of increased dietary intake, noting that “High fecal potassium losses … in all patients probably helped maintain normal serum potassium concentrations.” A plant-based diet, which is high in fiber, may also augment K^+^ excretion via increased stool volume, further attenuating a potential rise in serum K^+^, whereas diets high in potassium from animal sources or chemical additives may not have this effect. Further research is needed to investigate this speculative benefit.

### 3.2. Phosphorus

As with other CKD patients, hyperphosphatemia is a concern in those on PD, although data regarding phosphorus levels and outcomes are surprisingly scant. Several large epidemiologic studies have shown that phosphorus levels ≥6.4 mg/dL (PD and HD) [85], ≥6.5 mg/dL (PD only) [86], or ≥7 mg/dL (PD only) [87] are associated with all-cause mortality, although no studies have shown that intervention improves outcomes. Despite this, most nephrologists do treat hyperphosphatemia, and the 2017 Clinical Practice Guideline Update for the Diagnosis, Evaluation, Prevention, and Treatment of Chronic Kidney Disease–Mineral and Bone Disorder (CKD-MBD) guidelines recommend (albeit with weak evidence) “lowering elevated phosphate levels toward the normal range” for all CKD patients, including those on dialysis [88]. In an effort to control serum phosphorus, dialysis patients are often advised to restrict their dietary intake of phosphorus [88] and prescribed phosphorus binders. The Peritoneal Dialysis Outcomes and Practice Patterns Study (PDOPPS) showed that approximately 75% of individuals using PD worldwide use phosphorus binders [87]. In addition to the risk of adverse events [89] (mainly gastrointestinal or, in the case of calcium binders, hypercalcemia), phosphorus binders account for almost half of the pill burden for those on PD [90]. Concurrently, people on dialysis are also often counselled to eat high-protein foods, which tend to contain a lot of phosphorus, leading to potential confusion about what to eat and frustration at receiving conflicting information. Despite these efforts, serum phosphorus remains 5.5 mg/dL or above in approximately 37% of PD patients [87].

Over the last decade, the source of phosphorus in CKD patients has received significant attention. The recent Kidney Disease Improving Global Outcomes (KDIGO) guidelines also recommend that the source of phosphorus be considered [88]. It is clear that plant-based phosphorus, by virtue of its inclusion in phytate (which humans cannot readily digest due to the absence of the degrading enzyme phytase), is less absorbable than animal protein, whereas inorganic phosphate added during food processing is nearly completely absorbed [91], although phosphorous bioavailability in plant-based foods can increase depending on processing and preparation methods [92].

In a crossover feeding study in individuals with non-dialysis dependent CKD, Moe et al. [93] showed that for a given load of dietary phosphorus, plant protein leads to lower phosphorus levels than animal protein, although this type of metabolic study has not been conducted in those on PD. In their cohort study of the PD population, Liu et al. [20], found no difference in serum phosphorus in the highest tertile of the plant protein intake compared with the lowest. To date there have been no interventional trials comparing the effects of animal vs. plant protein on serum phosphorus levels in individuals on PD.

### 3.3. Energy and Protein Intake

PD patients are at high risk for protein energy wasting and undernutrition. A recent meta-analysis found that the median prevalence of protein energy wasting in those on PD was 36% [94]. There are concerns that plant-based diets will not supply enough protein, high quality protein, and/or energy, particularly for those who are strictly vegan.

A recent meta-analysis found that in the general population, while strict vegans did have the lowest total energy intake when compared with other dietary patterns, typically they do meet the recommended daily intake [95]. On average, those consuming a vegan diet also meet their recommended daily protein intake, defined as 0.8 g/kg [96] or >10% of total calories [97], with mean protein intakes averaging approximately 13–14% of the total caloric intake [95,98]. Studies out of Denmark and Belgium showed that vegans consume a mean of 75.5 and 82 g of protein per day compared with 94 and 112 g/day in the general population and meat eaters, respectively, although it must be noted that a small percentage of vegans may not achieve their daily recommended protein intake [24,25].

Another common concern is that plant-based protein is of a lesser biologic value compared with animal protein and that those consuming a primary plant-based diet may not ingest adequate amounts of essential amino acids. While this may be true if the diet does not provide adequate energy or is extremely restrictive and limited to just one or two food sources, this is not an issue in those consuming a varied plant-based diet with an adequate number of calories [98,99]. To date, there are no data showing that those consuming a vegan diet providing adequate energy experience any adverse effects from a protein deficiency or deficiency of any specific amino acid.

Data regarding protein intake and albumin levels in predominantly plant eaters are limited in those on dialysis. Some studies have noted lower albumin levels in vegetarians vs. non vegetarian dialysis patients [100,101], whereas others have not shown a relationship between plant protein intake and serum albumin [20,102]. In their cohort study of 884 PD patients, Liu et al. found that those in the highest tertile of the percent of protein intake from plants had higher albumin levels than those in the lowest tertile, despite lower total protein intake. Interestingly, they also had a higher energy intake as well [20]. Whether diets high in plant protein and those high in animal protein provide equivalent nutrition for those on PD deserves further study.

Protein homeostasis is of particular importance in PD, where there is the added issue of peritoneal protein loss. Albumin loss across the PD membrane averages approximately 5–8 g per day [103], and normally, this loss would be compensated by increased albumin synthesis by the liver, a process which is suppressed by inflammation [104] and chronic acidosis [105]. It is possible (but unproven) that the decrease in inflammation and improvement in acidosis afforded by more plant-based diets may compensate for, or even outweigh, the potential decrease in total protein intake. This deserves further investigation as well.

### 3.4. Other Considerations

Vitamin D deficiency is common in those on PD [106], and this may be an additional concern in strict vegans [107]. This concern is not trivial, as vitamin D deficiency in the PD population has been associated with numerous adverse consequences [108,109,110], including peritonitis [111]. Since Vitamin D deficiency is so common, careful monitoring and intervention are required in all individuals on PD, irrespective of dietary patterns. Vitamin B-12 is absent from a plant-based diet, and any individual consuming such a diet should take a supplement. Studies have also shown that vegans may have lower selenium, vitamin A, and iodine levels than non-vegans, although the clinical significance of this is not certain [25].

**Figure 1:**
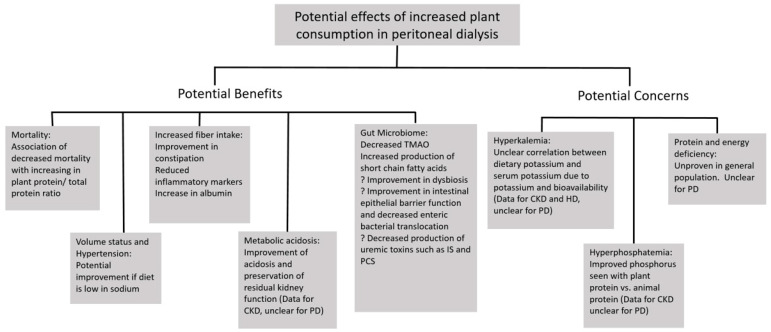
Potential benefits and concerns of increased plant consumption in peritoneal dialysis. CKD: Chronic kidney disease, PD: Peritoneal dialysis, HD: hemodialysis, TMAO: trimethylamine-N-oxide, IS: indoxyl sulfate, and PCS: p-cresol/p-cresyl sulfate.

Figure 1 summarizes the potential advantages and disadvantages of plant-based diets in peritoneal dialysis.

## 4. Conclusions

Plant-based diets may benefit individuals on peritoneal dialysis. They have been associated with improved mortality, and they may help mitigate issues common in those on PD, such as constipation, volume and sodium overload, hypertension, and metabolic acidosis, as well as exert beneficial effects on the gut microbiome. While there are concerns regarding the effects of a plant-based diet on total energy and protein intake; malnutrition; and serum potassium, phosphorus, and albumin, these have not been borne out by available data. More research is needed to determine whether the potential benefits of plant-based diets will correspond to improved outcomes in the peritoneal dialysis population and whether the potential disadvantages are truly a clinical concern.

## Data Availability

Not applicable.
